# Metabolic resistance drives cross-resistance in *Aedes albopictus* to pyriproxyfen and pyrethroid insecticides

**DOI:** 10.1371/journal.pone.0355196

**Published:** 2026-08-31

**Authors:** Xuexue Kong, Dan Wang, Jingzhu Zhou, Dijun Chen, Xiaoyi Lu, Ting Lei, Yong Hu, Wenqin Liang

**Affiliations:** 1 School of Public Health, the Key Laboratory of Environmental Pollution Monitoring, and Disease Control, Ministry of Education, Guizhou Medical University, Guiyang, People’s Republic of China; 2 Department of Vector Surveillance Section of Guizhou Center for Disease Control and Prevention, Key Laboratory of Microbiome and Infectious Disease Prevention and Control, Guiyang, People’s Republic of China; Advanced Centre for Chronic and Rare Diseases, INDIA

## Abstract

*Aedes albopictus* is a major global vector of arboviruses. Pyriproxyfen, an insect growth regulator, is increasingly used for mosquito control, but the potential for resistance and cross-resistance with pyrethroids poses a serious threat. Through 13 generations of laboratory selection, we established a highly pyriproxyfen-resistant *Ae. albopictus* strain (Lab-R, RR_50_ = 10.12). This strain developed moderate to high cross-resistance to the permethrin, deltamethrin, and beta-cypermethrin (RR_50_ = 8.11–10.30). Resistance was associated with significant fitness costs, including prolonged larval development, reduced female longevity, and lower fecundity and egg hatching. Crucially, resistance of Lab-R was driven by enhanced metabolic enzyme activity (P450s, GSTs, Car Es), with no target-site mutations detected in the CHS-1 or kdr genes. Synergist assays confirmed that enzyme inhibitors largely restored insecticide susceptibility. These findings demonstrate that metabolic resistance mediates cross-resistance between pyriproxyfen and pyrethroids in *Ae. albopictus*, carrying substantial fitness trade-offs. For vector control programs, this warns against the overreliance on rotational or sequential use of pyriproxyfen and pyrethroids as the sole rotation strategy, emphasizes the need for routine monitoring of metabolic resistance, and supports the potential use of synergists such as PBO to restore efficacy where resistance has emerged.

## 1. Introduction

*Aedes albopictus*, also known as the Asian tiger mosquito, is a highly invasive and significant vector species originating from Southeast Asia. It is now widely distributed across all continents except Antarctica and is listed among the world’s most dangerous invasive species [1,2]. This mosquito exhibits high aggressiveness and can carry and transmit multiple pathogens including dengue virus, Zika virus, and chikungunya virus, posing a severe threat to public health [1,3,4]. Among these, dengue fever, as the most rapidly spreading mosquito-borne viral disease, has high incidence rates and readily triggers large-scale epidemics, resulting in substantial disease and economic burdens [5–7]. Currently, due to the lack of specific drugs and vaccines, chemical insecticide application remains the primary method for controlling mosquito populations and preventing mosquito-borne diseases [8]. Pyrethroid insecticides are widely used due to their high efficacy and low toxicity to mammals. However, prolonged and improper application has led to widespread and severe insecticide resistance among mosquitoes [9,10].

The increasing resistance of mosquitoes to traditional chemical insecticides has become a critical factor limiting control effectiveness. Therefore, there is an urgent need to develop and apply novel insecticides with different modes of action [11,12]. Pyriproxyfen (PPF), as a juvenile hormone analog, exhibits a unique mode of action by interfering with metamorphosis and adult emergence, thereby reducing mosquito populations without acute toxicity to non-target organisms [13–15]. Owing to these characteristics, the World Health Organization (WHO) has recommended PPF for use in integrated vector management, particularly in larvicidal and autodissemination strategies [16,17]. However, recent field studies have increasingly reported reduced susceptibility to PPF in *Ae. albopictus* populations from Brazil [18,19], Malaysia [20,21], the United States [22,23], and China [24,25]. These findings indicate that resistance to PPF is emerging even under its relatively novel mechanism, posing a direct challenge to WHO-recommended control strategies and underscoring the urgent need to elucidate the resistance mechanisms and associated fitness costs in *Ae. albopictus*. The mechanisms underlying insecticide resistance in mosquitoes are complex, primarily encompassing metabolic resistance, target site resistance, penetration resistance, and behavioral resistance [26–28]. Among these, metabolic resistance refers to increased activity or expression of detoxification enzyme systems (primarily including cytochrome P450 monooxygenases, glutathione S-transferases, and carboxylesterases) within the insect’s body, thereby accelerating the metabolism of insecticides [29,30]. Research indicates that in the malaria vector *Anopheles gambiae*, the overexpression of P450s is closely associated with resistance to pyriproxyfen and pyrethroid insecticides [31]. Molecular docking analysis suggests that P450 subtypes such as CYP2C19, CYP2C9, and CYP3A4 in wild populations of *Ae. albopictus* from Fujian Province, China, may possess the potential to metabolize pyriproxyfen [25]. Furthermore, previous studies have identified cross-resistance between pyriproxyfen and deltamethrin [24,25,32], though the underlying molecular mechanisms remain unclear.

Although previous studies have documented pyriproxyfen resistance in *Ae. albopictus* from China, Brazil, and Malaysia, they primarily focused on resistance monitoring or limited mechanistic analyses (e.g., P450 expression or molecular docking). To date, no study has systematically integrated long-term laboratory selection of PPF resistance, comprehensive fitness cost assessment, and cross-resistance mechanisms with pyrethroids in a single *Ae. albopictus* model. Specifically, the following key questions remain unanswered: (1) how PPF resistance develops over generations under sustained selective pressure; (2) whether PPF resistance imposes detectable fitness costs (e.g., effects on fecundity, longevity, or development); and (3) the molecular basis underlying cross-resistance between PPF and pyrethroid insecticides, which has been observed but not mechanistically validated. Therefore, this study aims to fill these gaps by establishing a PPF-resistant strain of *Ae. albopictus* through laboratory screening, followed by integrated physiological, biochemical, and molecular analyses. The findings will provide novel insights into resistance evolution and cross-resistance mechanisms, offering a scientific basis for optimizing resistance management strategies distinct from previous geographically limited or single-faceted investigations.

Therefore, this study aims to comprehensively investigate the development patterns of pyriproxyfen resistance in *Ae. albopictus*, the fitness costs associated with resistance, and the mechanisms underlying cross-resistance with pyrethroid insecticides. This will be achieved through systematic laboratory screening of pyriproxyfen-resistant strains, combined with physiological, biochemical, and molecular biological approaches. The findings are expected to provide theoretical support and practical guidance for the scientific control and resistance management of *Ae. albopictus*.

## 2. Materials and methods

### 2.1. Ethics statement

This study involved the use of mice for blood feeding of mosquitoes. All animal procedures were approved by the Institutional Animal Care and Use Committee of Guizhou Center for Disease Control and Prevention (Approval No. DS2025−02).

### 2.2. Mosquito strains and selection of Pyriproxyfen-Resistant strain (Lab-R)

The *Ae. albopictus* sensitive strain (Lab-S) used in this study was obtained from the National Institute for Communicable Disease Control and Prevention, Chinese Center for Disease Control and Prevention. It has been maintained long-term in the vector breeding facility of the Guizhou Provincial Center for Disease Control and Prevention without exposure to any insecticides. Using Lab-S *Ae. albopictus* as the base population, continuous resistance screening was conducted via larval immersion to establish the laboratory pyriproxifen-resistant strain (Lab-R). Rearing conditions were as follows: temperature 27 ± 1°C, relative humidity 70 ± 5%, and a photoperiod of 14L: 10D. Larvae were fed a specialized diet (pig liver powder: steamed bun powder = 1: 1), while adults were provided 10% glucose water. Mouse blood meals were administered 3–5 days post-eclosion to stimulate oviposition. The median inhibitory concentration (IE_50_) of pyriproxyfen was determined using the larval immersion method recommended by the WHO [33]. For each generation, late-III to early-IV instar larvae were selected and exposed to a pyriproxyfen solution at their contemporary IE_50_ concentration. Two-thirds of each generation’s larvae underwent screening, while the remaining one-third were reared directly to adulthood for stock maintenance. If contemporary screening caused excessive larval mortality hindering passage, the next generation was bred from the stock adults for re-screening.

Control for confounding factors: When the control group’s emergence rate is 91%−99%, the emergence inhibition rate must be corrected using the Abbott formula for the treatment group: IE(%) = (Treatment mortality rate – Control mortality rate) ÷ (100 – Control mortality rate) × 100. If the emergence rate of the test insects in the control group is < 90%, the experiment must be repeated [33].

### 2.3. Cross-resistance assay

The larval immersion test was used to the determine resistance of Lab-R strain larvae to three pyrethroid insecticides (permethrin, deltamethrin, and beta-cypermethrin, all provided by the Institute for Infectious Disease Prevention and Control, Chinese Center for Disease Control and Prevention) [33]. The technical insecticide was dissolved in acetone to prepare a 3 mg/mL stock solution, which was then serially diluted into 5–8 concentration series. A 0.1 mL aliquot of each solution was added to a beaker containing 199.9 mL of dechlorinated water and mixed thoroughly. Three replicates were set for each concentration, with 20 late III-stage to early IV-stage Lab-R strain larvae placed in each replicate. A solvent control group was also included. After 24 hours, record mortality based on lack of response to touch or tail tremors only. If control mortality < 5%, no correction is needed; for mortality between 5%−20%, apply the Abbott formula; if control mortality > 20% or pupation rate > 10%, repeat the experiment.

### 2.4. Fitness cost assay

The sample size and experimental design were primarily based on the study by Deng J et al. [34] and their corresponding thesis, which systematically assessed the fitness costs of insecticide-resistant *Ae. albopictus*.

#### 2.4.1. Life table at larvae stage.

Egg papers from Lab-S and Lab-R strains were hatched. Fifty randomly selected first-instar larvae from each strain were reared under identical conditions. Larval pupation, pupal emergence, and mortality were observed and recorded daily until complete emergence or death. Four replicates were established for each strain.

#### 2.4.2. Adult mosquito survival.

Twenty-five female and twenty-five male adult mosquitoes from the Lab-S and Lab-R strains, hatched on the same day, were reared separately in mosquito cages. Daily mortality counts were recorded, and 10% glucose water was replenished until all test insects died. Four replicates were conducted for each strain.

#### 2.4.3. Fecundity and hatching rate.

Forty female and fifteen male mosquitoes from the Lab-S and Lab-R strains, respectively, with similar emergence times, were placed in mosquito cages. They were fed 10% glucose solution daily. On the fourth day, after blood feeding to satiety, females were housed individually and provided with oviposition filter paper. After 7 days, count the eggs laid by each female (including any hatched larvae). Place the filter paper in dechlorinated water for one week of incubation, then record the number of hatched larvae to calculate the hatching rate.

#### 2.4.4. Measurement of adult mosquito size.

Female mosquitoes from Lab-S and Lab-R strains were cryo-anesthetized and a super-depth-of-field microscope (Carl Zeiss Microscopy GmbH, Germany, Smartzoom5) wing length was measured under from the membranous axillary lobe at the wing margin base to the wing tip. Ten individuals per strain were measured. Additionally, female mosquitoes that emerged on the same day were dried and in groups of 10 (n = 4) using an analytical balance weighed.

### 2.5. Metabolic enzyme activity assay

This study focused on cytochrome P450 monooxygenases (P450s), glutathione S-transferases (GSTs), and carboxylesterases (Car Es), as enhanced or overexpressed activity of these enzymes correlates with insecticide resistance [35]. Enzyme activities of P450s, GSTs, and Car Es in Lab-R and Lab-S strain larvae were measured using a microplate reader (Thermo Scientific, USA, Multiskan FC) and the sample size was determined based on the study by Tokponnon TF et al [36–38]. Eighty late III-stage to early IV-stage larvae each from Lab-S and Lab-R strains were ground in PBS buffer, centrifuged at 4°C, 15,000 r/min for 15 min, and the supernatant collected as the enzyme source. Protein concentration was determined according to the BCA Protein Assay Kit (Thermo Scientific, USA) protocol.

P450s activity: Employed the 3,3’,5,5’-tetramethylbenzidine (TMBZ) (Sigma, USA) colorimetric assay. Measured absorbance at 630 nm and indirectly calculated P450s content using a standard curve of cytochrome C (China National Institute for Food and Drug Control, China).

GSTs activity: Using 1-chloro-2,4-dinitrobenzene (CDNB) (Sigma-Aldrich, USA) as substrate, measure absorbance change at 340 nm over 10 min reaction time. Enzyme activity was calculated based on CDNB’s extinction coefficient.

Car Es activity: Using α-acetylnaphthol (Sigma, USA) as substrate, the reaction was visualized with Gentian Blue B salt (Shanghai Yuanye Biotechnology Co., Ltd., China). Absorbance was measured at 595 nm, and enzyme activity was calculated using an α-acetylnaphthol (Sigma, USA) standard curve.

### 2.6. Synergist experiment

Pyriproxyfen, permethrin, deltamethrin, and beta-cypermethrin were mixed with enzyme inhibitors [piperonyl butoxide (PBO), diethyl maleate (DEM), and tris(phenyl) phosphate (TTP)] in 1: 1, 1: 3, 1: 5 ratios to prepare stock solutions, with concentrations calculated based on insecticide content. Larval toxicity to Lab-R strain larvae was assessed via larval immersion [33], and synergist ratios (SR) were calculated.

### 2.7. DNA extraction and gene mutation detection

Two female mosquitoes constituted one sample. Genomic DNA was extracted using a kit (Beijing BioTec Biotechnology Co., Ltd., China). Specific primers were designed for PCR amplification targeting the chitin synthase gene (CHS-1) and the knock-down resistance gene (kdr) F1534 site (Table 1). Amplification products were verified by agarose gel electrophoresis and sent to Shanghai Bioengineering Co., Ltd. for single-direction sequencing. Mutations were analyzed through sequence alignment.

**Table 1:** Genomic DNA amplification primers.

Gene	Primer sequence	PCR product size (bp)	Reference
CHS-1	kkv F3: 5’-TCG GAA GTC CTT CGG CTT ATT C-3’	350	[39]
	kkv R3: 5’-TGG ATA CTT CAA TGG AAC CTT CC-3’		
1534	aegSCF7: 5’-GAG AAC TCG CCG ATG AAC TT-3’	740	[40]
	aegSCR7: 5’- GAC GAC GAA ATC GAA CAG GT-3’		

### 2.8. Statistical analysis

SPSS Statistics 31.0 software was used to calculate IE_50_/LC_50_ values and their 95% confidence intervals via Probit analysis. Resistance ratio (RR_50_) = IE_50_/LC_50_ of screened strain ÷ IE_50_/LC_50_ of sensitive strain. Resistance level classification: when RR_50_ was < 5 the popilation was considered susceptible, when RR_50_ was between 5 and 10 were considered to have moderate resistant, and when RR_50_ is ≥ 10 the mosquitos were highly resistant [33]. Synergist ratio (SR) = IE_50_/LC_50_ without synergist ÷ IE_50_/LC_50_ with synergist.

Continuous variables such as larval development time, wing length, and body weight are expressed were tested for normality using the Shaprio-Wilk test. For normally distributed data with homogeneous variance (Levene’s test, *P* > 0.05), comparisons between Lab-S and Lab-R strains were performed using two-tailed independent-sample t-tests. When variances were unequal (Levene’s test, *P* < 0.05), Welch’s t’-test was used. Categorical variables such as pupation rate, emergence rate, and hatching rate were compared using the *χ*^2^ tests. Adult survival curves were generated by the Kaplan–Meier method, and differences between strains were assessed by the log‑rank test, followed by the Mann–Whitney U test for pairwise comparisons of median survival time.

P450s, GSTs, and Car Es activity data were first tested for normality (Shapiro-Wilk test). If normally distributed and homoscedastic (Levene’s test, *P* > 0.05), a two‑tailed independent‑sample t-test was applied. If normally distributed but heteroscedastic (Levene’s test, P < 0.05), Welch’s t’-test was used. For data that did not follow a normal distribution, the Mann–Whitney U test was employed. Enzyme activity is expressed as mean ± SD or as *M*(*P*_25_-*P*_75_) accordingly. The significance level was set at *α* = 0.05.

Peak analysis and sequence alignment were performed on the measured sequences to observe mutations at each site, determine allele types, and establish genotypes.

## 3. Results

### 3.1. Selection and development patterns of resistant strains

**Fig 1:**
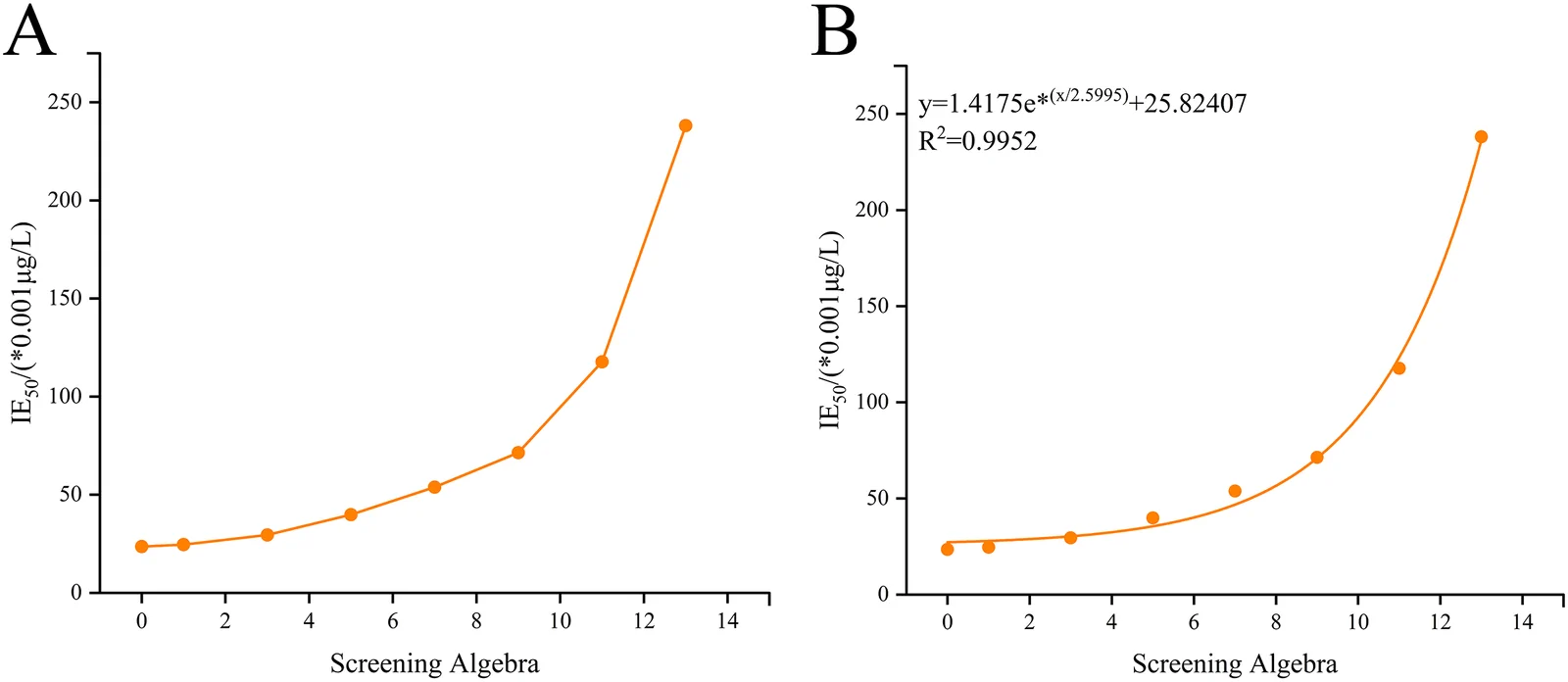
(A) Changes in larval IE_50_, (B) Scatter plot and fitting of larval IE_50_ changes.

After 13 generations of selection, the IE_50_ of pyriproxyfen against *Ae. albopictus* larvae increased from 23.542*0.001 μg/L to 238.138*0.001 μg/L, with a resistance ratio (RR_50_) of 10.12-fold (RR_50_ > 10-fold), successfully establishing a pyriproxyfen-resistant strain (Lab-R) of *Ae. albopictus* (Table 2). Resistance development followed an exponential growth pattern (y = 1.4175e*^(x/2.5995)^ + 25.8241, R^2^ = 0.9952), with slow accumulation during the first 11 generations (RR_50_ = 5.00) followed by rapid increase, reaching high resistance levels by generation 13 (Fig 1A, B).

**Table 2:** Changes in larval IE_50_.

Screening Algebra	IE_50_(95% *CI*) (*0.001 μg/L)	Slope	RR_50_
0	23.542 (18.010, 30.262)	1.299	1.00
1	24.601 (18.915, 31.581)	1.302	1.04
3	29.500 (22.882, 37.970)	1.315	1.25
5	39.816 (31.769, 49.751)	1.412	1.69
7	53.881(42.670,66.981)	1.434	2.29
9	71.406(57.229,88.849)	1.405	3.03
11	117.715 (96.525, 142.298)	1.663	5.00
13	238.138(197.429,286.535)	1.556	10.12

### 3.2. Cross-resistance of Lab-R strain to pyrethroid insecticides

**Fig 2:**
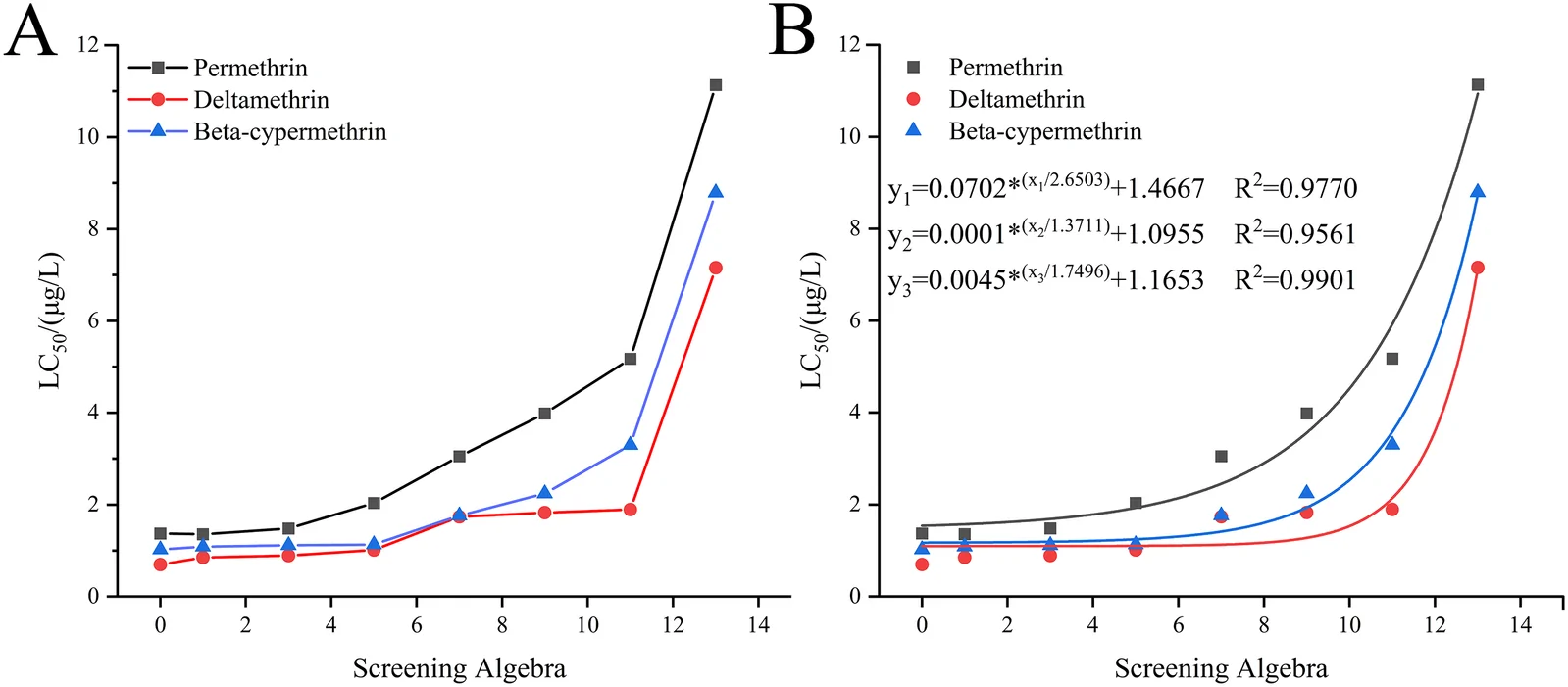
(A) Changes in larval LC_50_, (B) Scatter plot and fitting of larval LC_50_ changes, y1 is permethrin, y2 is deltamethrin, y3 is beta-cypermethrin.

After 13 generations of screening, the Lab-R strain developed significant cross-resistance to permethrin, deltamethrin, and beta-cypermethrin, with LC_50_ values increasing to 11.134 μg/L, 7.156 μg/L, and 8.789 μg/L, respectively. With RR_50_ values of 8.11-, 10.30-, and 8.58-fold, respectively, showing high resistance to deltamethrin (Fig 2A). Resistance development for all three pyrethroid insecticides followed an exponential growth model (Fig 2B).

### 3.3. Fitness cost

#### 3.3.1. Developing time in Lab-R much longer than Lab-S.

**Fig 3:**
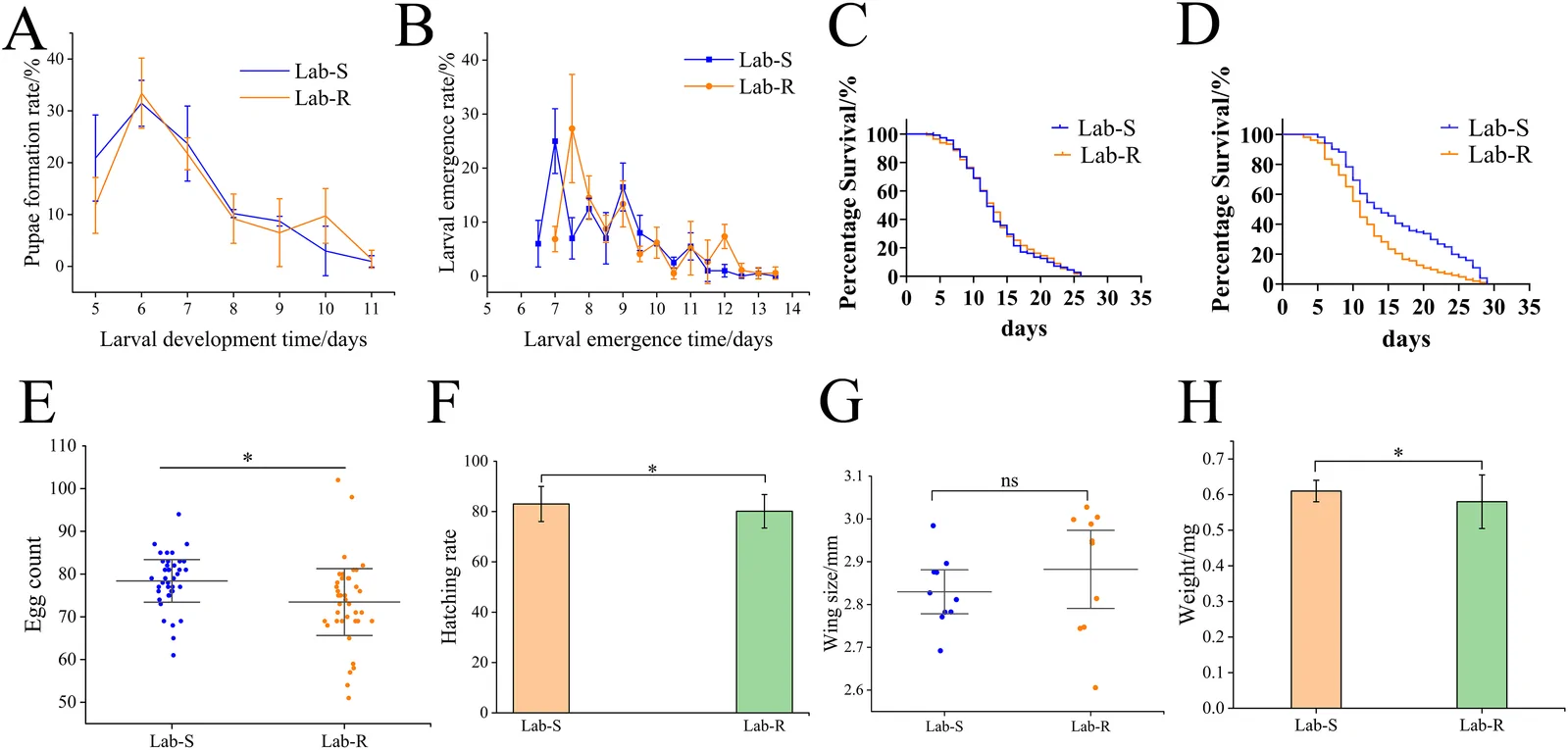
Life table analysis of susceptible and resistant strains of *Ae. albopictus.* (A, B) represent larval development duration, (C, D) indicate survival time for males and females, respectively, (E, F) denote egg production and hatching rate, respectively, (G, H) reflect wing length and body weight of females, respectively. *P < 0.05, ns: no significance.

The pupation rate (93.74% vs 99.05%) and emergence rate (87.36% vs 97.13%) of the Lab-R strain were lower than those of the Lab-S strain, but only the emergence rate showed a significant difference (*P* < 0.05). The total developmental time from larva to pupa, pupa to adult, and the entire larval period were significantly longer in the Lab-R strain than in the Lab-S strain (*P* < 0.05) (Table 3, Fig 3A and B).

**Table 3:** Developmental duration of *Ae. albopictus* Lab-S and Lab-R dtrains from larva to adult.

Evaluation Indicators	Lab-S strain	Lab-R strain
Pupation rate (%)	99.05 ± 1.10	93.74 ± 3.00
Eclosion rate (%)	97.13 ± 2.44	87.36 ± 6.20
Larva-Pupa development time (d)	6.67 ± 1.39	6.99 ± 1.55
Pupa-Adult development time (d)	1.81 ± 0.38	1.91 ± 0.41
Larval-adult development time (d)	8.46 ± 1.47	8.87 ± 1.62

#### 3.3.2. Survival time in Lab-R much shorter than Lab-S.

The mean survival time (12 d vs. 16 d) and median survival time (11 d vs. 14 d) of Lab-R female mosquitoes were significantly shorter than those of Lab-S mosquitoes (*P* < 0.0001). No significant difference in survival time was observed between the two strains in males (Fig 3C, D).

#### 3.3.3. Fecundity and egg hatching rate in Lab-R lower than Lab-S.

The single-female egg production (73 vs. 78 eggs) and egg hatching rate (80.39% vs. 83.20%) of Lab-R were significantly lower than those of Lab-S (*P* < 0.05) (Fig 3E, F).

#### 3.3.4. Female Lab-R and Lab-S strains differ significantly in weight but not in wing length.

Under identical rearing conditions with equal food availability, wing length showed no statistically significant difference between Lab-S and Lab-R female mosquitoes. However, Lab-R females exhibited significantly lower body weight (0.58 mg vs. 0.61 mg) compared to Lab-S females (*P* < 0.0001) (Fig 3G, H).

### 3.4. Metabolic detoxification enzyme activities

**Fig 4:**
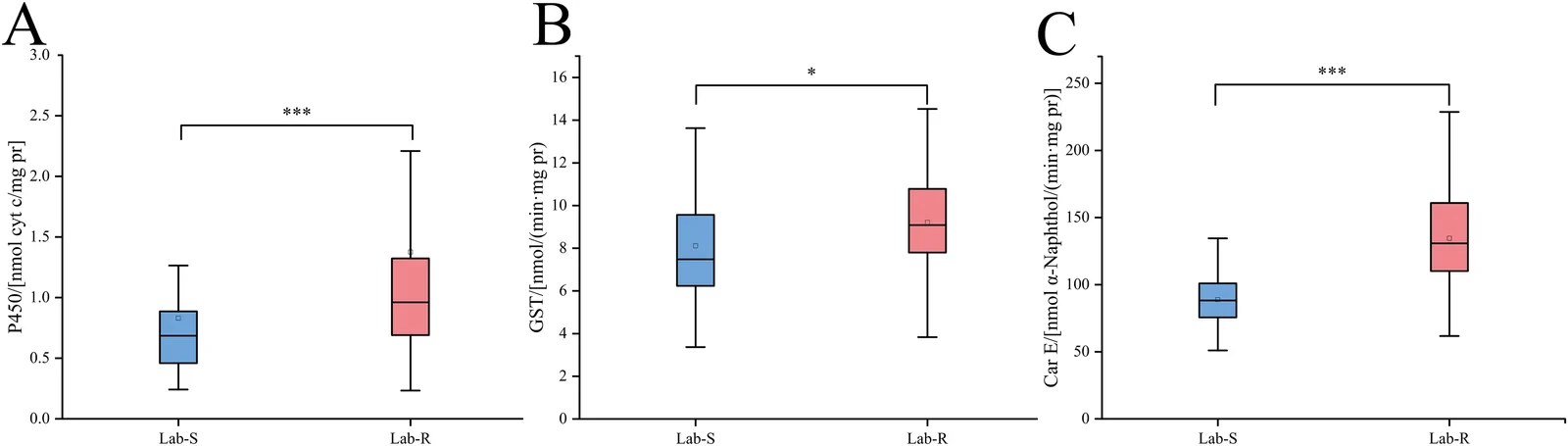
Enzyme activities of (A) P450s, (B) GSTs, and (C) Car Es in larvae of the Lab-S and Lab-R *Ae. albopictus* susceptible strain and pyriproxyfen-resistant strain, *P < 0.05.

Compared to the Lab-S strain, the activity of three major metabolic detoxification enzymes was significantly elevated in the larvae of the Lab-R strain. Specifically, P450s activity was 1.42-fold higher (*P* < 0.0001), GSTs activity was 1.22-fold higher (*P* < 0.05), and Car Es activity was 1.48-fold higher (*P* < 0.0001) than in Lab-S larvae (Fig 4).

### 3.5. Synergist boosts Lab-R Ssusceptibility to pyriproxyfen and pyrethroids

**Fig 5:**
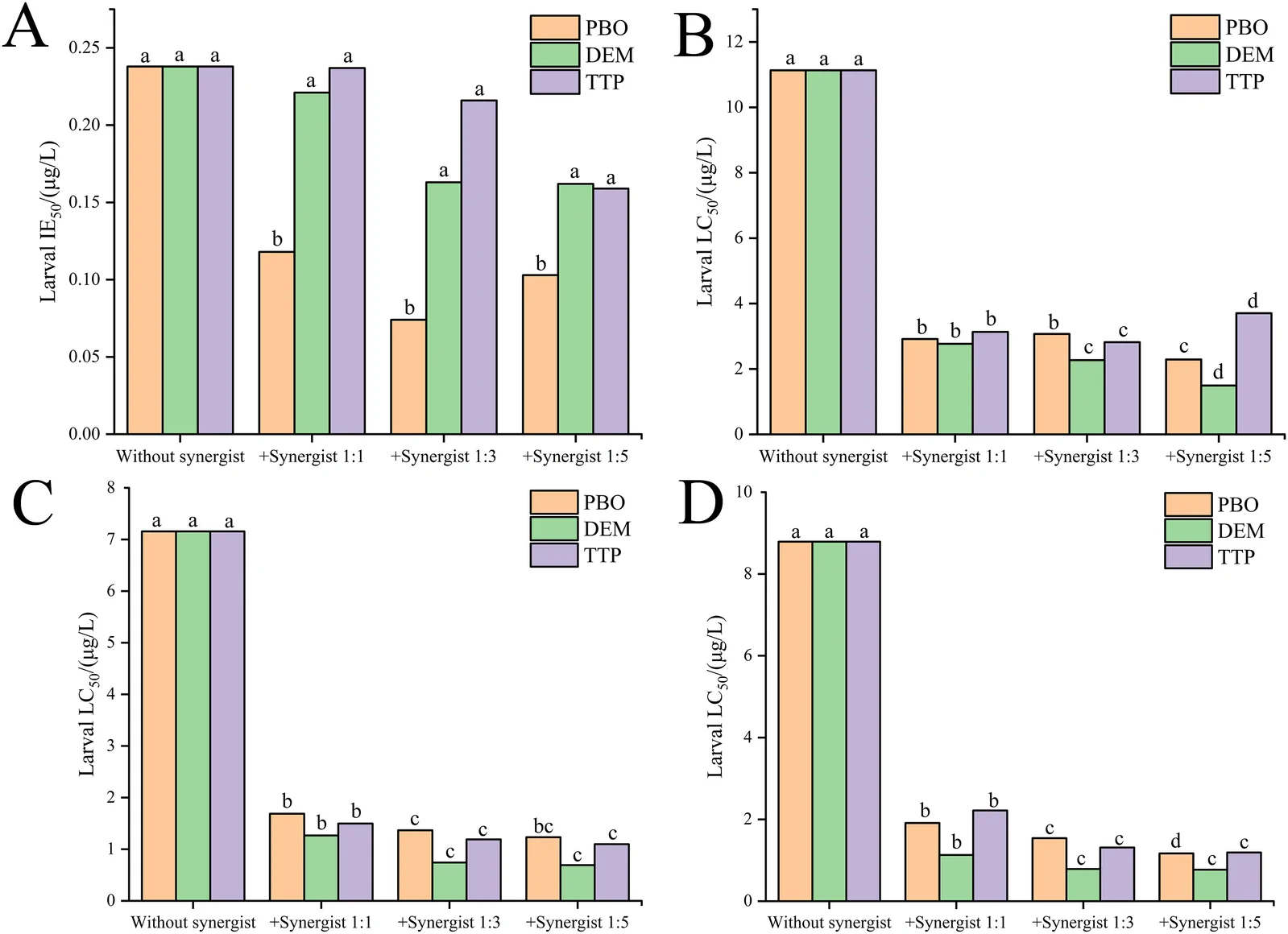
Effects of different synergists on IE_50_/LC_50_ values for laboratory-raised *Ae. albopictus* Larvae. A is pyriproxyfen, B is permethrin, C is deltamethrin, D is beta-cypermethrin.

The three enzyme inhibitors (PBO, DEM, and TTP) exhibited no significant toxicity to Lab-R larvae when used alone. When combined with insecticides, they demonstrated varying degrees of synergistic effects (Fig 5).

For pyriproxyfen: PBO exhibited the strongest synergistic effect (3.22-fold increase at SR 1: 3), DEM showed weaker but increasing effects with higher ratios, and TTP only produced a significant synergistic effect at a 1: 5 ratio (SR = 1.50) (Fig 5A).

For pyrethroid insecticides: PBO, DEM, and TTP exhibited significant and potent synergistic effects with all three pyrethroid insecticides. Among these, DEM produced the highest synergistic ratios for deltamethrin and cypermethrin, reaching 10.34-fold and 11.18-fold, respectively (Fig 5B, C, D).

### 3.6. Lab-R target site mutation-free

Analysis of sequencing results from 80 samples revealed no mutations at the I1043 site of chitin synthase CHS-1 or the F1534 site of the kdr gene.

## 4. Discussion

Through systematic laboratory screening, this study successfully obtained a Lab-R strain of *Ae. albopictus* exhibiting high-level resistance to pyriproxyfen (RR_50_ = 10.12). Notably, Lab-R strain larvae exhibited moderate to high cross-resistance to three commonly used pyrethroid insecticides (RR_50_: 8.11–10.30), consistent with findings of pyriproxyfen resistance in deltamethrin-resistant *Ae. albopictus* strains [24]. This finding carries significant practical implications: it indicates that even without direct pyriproxyfen exposure, wild *Ae. albopictus* populations in regions with severe pyrethroid resistance may develop potential pyriproxyfen resistance due to cross-resistance mechanisms, and vice versa. This serves as a warning for insecticide rotation and mixing strategies in public health practice.

Resistance development often carries fitness costs. This study found that the Lab-R strain exhibited disadvantages across multiple life history traits: prolonged larval development, reduced female lifespan, decreased fecundity, and lower hatching rates. These findings align with previous research on cyfluthrin-resistant *Ae. albopictus* strains showing extended development times and shortened lifespans [34]. These findings align with studies on *Anopheles gambiae* exposed to pyriproxyfen-treated bed nets [41] and *Ae. albopictus* treated with sublethal pyriproxyfen doses [42], indicating that pyriproxyfen can impair mosquito reproductive capacity and egg hatching rates. Such alterations may weaken the competitive ability of resistant populations in environments without insecticide selection pressure, theoretically providing a potential time window for resistance management.

Regarding resistance mechanisms, this study provides multifaceted evidence collectively pointing to the central role of metabolic resistance. First, activity levels of three key detoxification enzyme systems—P450s, GSTs, and Ca Es—were significantly elevated in the Lab-R strain. Second, corresponding enzyme inhibitors (PBO, DEM, TTP) [43–46] significantly reversed the Lab-R strain’s resistance to pyriproxyfen and all tested pyrethroid insecticides. Notably, PBO (a P450 inhibitor) exhibited the most pronounced and widespread synergistic effects across all compounds, strongly suggesting that P450 enzymes play a pivotal role in mediating this cross-resistance.This aligns with prior findings in Anopheles gambiae, where certain P450 enzyme families (e.g., CYP6P, CYP9J subfamilies) metabolize both pyriproxyfen and multiple pyrethroids [31,32]. The absence of mutations at the CHS-1 I1043 and kdr F1534 loci in this study further excludes the contribution of target site resistance in the screened strains, establishing metabolic resistance as the primary resistance mechanism.

The innovation of this study lies in systematically confirming that elevated metabolic detoxification enzyme activity serves as common mechanism underlying *Ae. albopictus* resistance to pyriproxyfen and cross-resistance to pyrethroid insecticides. However, limitations exist, such as the failure to identify specific key metabolic genes (P450s, GSTs, or Car Es) and the lack of validation of their expression changes at the transcriptional level.

In summary, *Ae. albopictus* can develop high-level resistance to pyriproxyfen under laboratory screening conditions, a process that is accompanied by significant fitness costs. More importantly, this resistance is primarily mediated by enhanced activity in three metabolic detoxification enzyme systems: P450s, GSTs, and Car Es. This mechanism also leads to cross-resistance to permethrin, deltamethrin, and beta-cypermethrin. Notably, mutations in the target genes CHS-1 and kdr (F1534) do not contribute to this resistance process. Based on these findings, we recommend that *Ae. albopictus* control programs avoid long-term monotherapy with pyriproxyfen or rotational use with other pyrethroid insecticides of the same class involved in this study to prevent the rapid development of cross-resistance. Additionally, monitoring metabolic enzyme activity should be incorporated into resistance surveillance systems, and the use of synergists such as PBO should be considered to restore insecticide efficacy. Future research should focus on utilizing omics technologies such as transcriptomics and proteomics to precisely identify key metabolic genes mediating this cross-resistance, thus providing targets for developing novel resistance molecular markers and formulating precision resistance management strategies.
