# Supplementary material for: Metabolic resistance drives cross-resistance in Aedes albopictus to pyriproxyfen and pyrethroid insecticides
Source: PLoS One. 2026 Aug 31;21(8):e0355196. doi: 10.1371/journal.pone.0355196 (PMC13528961; doi:10.1371/journal.pone.0355196)
Supplement: Supplementary file 1 [file pone.0355196.s002.docx]

Response to Reviewers

Dear editor and reviewers,

We have revised the manuscript and answered the editor and reviewer' s questions/comments with point by point hereunder according to the requirements in your letter.

Hence, we are re-submitting herewith the revised manuscript entitled ''Metabolic Resistance Drives Cross-Resistance in *Aedes albopictus* to Pyriproxyfen and Pyrethroid Insecticides'' and manuscript ID is PONE-D-26-02598.

To easily distinguish our answers from reviewer' s questions/comments, we highlighted all of our answers in blue while your letter and reviewer' s questions/comments in black in the Response letter.

Thank you again for your time and consideration. We hope the revised manuscript has now met the publication standard of your journal.

Looking forward to hearing from you.

**Journal's comments:**

1. Please ensure that your manuscript meets PLOS ONE's style requirements, including those for file naming.

Our response: Thank you for highlighting this. We have carefully reviewed and revised the manuscript's formatting, title, authors' affiliations, and file names in accordance with the template provided by PLOS ONE to ensure compliance with the journal's requirements.

2. Please clarify the role of funders in the study. Please note that funding information should not appear in the Acknowledgments section or other areas of your manuscript. We will only publish funding information present in the Funding Statement section of the online submission form. Please remove any funding-related text from the manuscript and let us know how you would like to update your Funding Statement.

Our response: Thank you for your feedback. We have removed all funding-related text from the manuscript, including the Acknowledgments section. The role of the funders was solely to provide financial support and they had no involvement in study design, data collection, analysis, interpretation, or manuscript preparation. Please find the updated Funding Statement as follows:

Funding Statement: This study was funded by Plan Project of the Science and Technology in Guizhou Province (No. Qian Ke He [2022] General 178), Guizhou Provincial Key Laboratory of Microbiome and Infectious Disease Prevention and Control (ZDSYS [2023] No. 004), and Science and Technology Fund Project of Guizhou Provincial Health Commission (No. gzwkj2025-504). The funders had no role in study design, data collection and analysis, decision to publish, or preparation of the manuscript.

This content has been removed from the manuscript; it has been highlighted in red and struck through.

3. Data Availability Statement.

Our response: Thank for your suggestion. All relevant data are within the manuscript and its Supporting Information files.

4. Please include your full ethics statement in the 'Methods' section of your manuscript file. In your statement, please include the full name of the IRB or ethics committee who approved or waived your study, as well as whether or not you obtained informed written or verbal consent.

Our response: We appreciate the editor's suggestion. We have added a complete ethics statement to the ‘Methods’ section (See Section 2.1 for details, text in red). This statement includes the full name of the review committee—the Animal Ethics Committee of the Guizhou Provincial Center for Disease Control and Prevention—and the approval number (DS2025-02).

5. Citations Recommended by the Reviewers.

Our response: Thank you for your feedback. The reviewer did not recommend any relevant references.

6. Completeness of the Reference List.

Our response: We are very grateful to the editor for raising this question.We have checked the reference list throughout the manuscript and confirmed that it does not contain any retracted publications.

7. As the study is important however, there are some observations on the Abstract lenght Justifying pyriproxyfen use as per WHO guidlines . in the methods the details for statistical methods used for enzyme activity analysis and fitness costs needs to added. modify the figures as per suggestions of the reviewers in result section and check overall MS for any repeatation. Improve discussion and ethical statements as suggested.

Our response: Thank you for your valuable comments on our manuscript. We have revised the manuscript accordingly.

Reviewer #1:

1.1 Consider shortening the abstract to focus on the most important findings, more explicitly highlight the broader implications for vector control programs.

Our response: Thank you for this valuable suggestion. We have simplified the abstract as suggested and included direct implications for vector control programs. Deleted text is indicated in red font with strikethrough. Added or revised text is highlighted in blue and underlined.

1.2 Strengthen the justification for focusing on pyriproxyfen by connecting it more directly to WHO recommendations and recent field reports. Clarify how this study offers something new compared to previous work in China, Brazil, and Malaysia.

Our response: We appreciate this important feedback. The introduction has been revised to incorporate recommendations from the World Health Organization (WHO) and the latest field reports on antimicrobial resistance from Brazil, Malaysia, the United States, and China, and to clarify how this study differs from previous studies conducted in China, Brazil, and Malaysia. Specific changes are highlighted in the file labeled 'Revised Manuscript with Track Changes. Deleted text is indicated in red font with strikethrough. Added or revised text is highlighted in blue and underlined.

1.3 Ensure ethical considerations (animal blood feeding, mouse use) are fully justified and referenced.

Our response: All procedures were conducted in accordance with the relevant provisions of the 'Guidelines on the Humane Treatment of Animals' and the 'Regulations on the Management of Laboratory Animals'. The ethics approval number is provided in Section 2.1 in the file labeled 'Revised Manuscript with Track Changes'.

1.4 Clarify how sample sizes were determined for each assay.
Our response: We thank the reviewer for this important question. The sample sizes in this study were determined based on established methodologies from previously published studies and international guidelines, as detailed below:

**Synergist experiments and resistance development:**

These experiments strictly followed the WHO guidelines for monitoring and managing insecticide resistance in *Aedes* mosquito populations (World Health Organization. Monitoring and managing insecticide resistance in *Aedes* mosquito populations. 2016.). For each concentration of insecticide (with or without synergist), three replicates were used, each containing 20 late-III to early-IV instar larvae. This sample size is the WHO-recommended standard for larval bioassays and has been widely adopted in insecticide resistance studies.

**Fitness Cost Experiment:**

The sample size and experimental design were primarily based on the study by Deng J et al.(Deng J, Guo Y, Su X, Liu S, Yang W, Wu Y, et al. Impact of deltamethrin- resistance in *Aedes albopictus* on its fitness cost and vector competence. PLoS Negl Trop Dis. 2021;15(4):e0009391.) and their corresponding thesis, which systematically assessed the fitness costs of insecticide-resistant *Ae. albopictus*. The sources for the experimental methods and sample size determination have been added to Section 2.4 of the manuscript. Added or revised text is highlighted in blue and underlined.

**Metabolic enzyme activity assays:**

The sample size and technical replicates were determined according to Tokponnon TF et al. (Tokponnon TF, Ossè R, Zoulkifilou SD, Amos G, Festus H, Idayath G, et al. Insecticide Resistance in *Aedes aegypti* Mosquitoes: Possible Detection of kdr F1534C, S989P, and V1016G Triple Mutation in Benin, West Africa. Insects. 2024;15(4):295.) and related studies on detoxification enzyme activity in mosquitoes. Each enzyme assay included three biological replicates per strain. This design ensures statistical reliability for enzyme activity comparisons. We have added the sample volume for the enzyme activity assay to Section 2.5 of the Methods section. Deleted text is indicated in red font with strikethrough. Added or revised text is highlighted in blue and underlined.

We have now explicitly cited these sources in the Methods section and clarified that sample sizes were based on established laboratory protocols rather than a formal power calculation, which we acknowledge as a limitation. No data-driven sample size re-estimation was performed during the study.

1.5 Provide more details on the statistical methods used for analyzing enzyme activity and fitness costs.

Our response: We thank the reviewer for this important methodological suggestion. We apologize for the lack of clarity. In the revised manuscript, we have substantially expanded the Statistical analysis section (section 2.8) to include:

Normality assessment – Shapiro-Wilk test for all continuous variables.

Variance homogeneity check – Levene's test before applying parametric tests.

Specific test choices: Two‑tailed independent t-test (homoscedastic) or Welch's t'-test (heteroscedastic) for normally distributed data; Mann-Whitney U test for non‑normally distributed data.

Survival analysis: Kaplan-Meier with log‑rank test and Mann–Whitney U for median survival.

Rate comparisons: χ² test for pupation, emergence, and hatching rates.

These changes are highlighted in the revised manuscript. Deleted text is indicated in red font with strikethrough. Added or revised text is highlighted in blue and underlined.

1.6 Present resistance ratios and enzyme activity data in clearer tables or figures for better readability.

Our response: We appreciate this constructive suggestion from the reviewers. To improve the readability of the manuscript, we have provided the raw data for the relevant figures and tables in the appendix. Please refer to the appendix for detailed results.

1.7 Discuss variability among replicates and confidence intervals.

Our response: Thank you for your careful reading and valuable comment regarding the variability among replicates and confidence intervals. In our manuscript, the variability among replicates is already reflected by the 95% confidence intervals (*CI*) provided for all IE_50_/LC_50_ values (Table 2, Fig 2), and standard deviations (*SD*) are presented for fitness cost data (Table 3, Fig 3). The number of replicates and statistical methods are described in the Materials and Methods section. Therefore, we believe the current results sufficiently convey the experimental variability without additional discussion. We kindly hope the reviewer agrees.

1.8 Consider adding statistical significance markers in the figures.

Our response: We thank the reviewer for this constructive suggestion. We would like to respectfully note that statistical significance markers (e.g., **P* < 0.05, ****P* < 0.0001, ns for non-significant and Identical letters within the same synergist group indicate that the difference is not statistically significant) have already been included in the relevant figures (Fig 3, Fig 4, and Fig 5) to indicate the results of statistical comparisons. To improve clarity and comply with the reviewer's expectation, we have now explicitly mentioned in the figure legends that asterisks denote statistically significant differences. We believe these revisions make the presentation of statistical results more transparent. We appreciate the reviewer's careful reading of our manuscript. Added or revised text is highlighted in blue and underlined.

1.9 Expand on the implications for integrated vector management, such as insecticide rotation and synergist use. Strengthen the call for policy and operational changes in vector control programs.

Our response: We thank the reviewer for the constructive comments. In response, we have expanded the discussion on the practical implications of our findings for integrated vector management (IVM), with a focus on insecticide rotation and the use of synergists such as PBO. We have also strengthened the policy and operational recommendations for vector control programs. For details, see the last paragraph of the discussion, deleted text is indicated in red font with strikethrough. Added or revised text is highlighted in blue and underlined.

1.10 Compare findings with resistance mechanisms in *Anopheles gambiae* and other vectors to broaden relevance.

Our response: Thank you very much indeed for your comment. We have added a comparative discussion on resistance mechanisms in *Anopheles gambiae* and other vectors (e.g., *Culex quinquefasciatus*, *Aedes aegypti*) in the Discussion section (see the highlighted section in light green). This broadens the relevance of our findings beyond *Ae. albopictus* and supports the conserved role of metabolic resistance across mosquito species.

1.11 Address limitations, including differences between laboratory strains and field populations, as well as geographic scope. Suggest future research directions, such as genomic studies and field validation.

Our response: We agree with the reviewer and have added the following points to the Discussion section:

Laboratory vs. field populations: We acknowledge that resistance development and fitness costs observed in a laboratory-selected strain may differ from wild populations due to genetic background and environmental factors.

Geographic scope: Our study was conducted on a single Ae. albopictus strain from one region; therefore, the findings may not be generalizable to all populations.

Future directions: We now recommend genomic studies (e.g., transcriptomics, whole-genome sequencing) to identify specific metabolic genes conferring cross-resistance, as well as field validation across multiple geographic sites to assess real-world applicability.

These revisions have been incorporated into the Discussion (see the highlighted section in light yellow) and deleted text is indicated in red font with strikethrough.

1.12 Ensure the funding statement includes grant numbers and URLs for funders. Confirm that funders had no role in study design or manuscript preparation.

Our response: Thank you for your careful and constructive comment regarding our funding statement. This work was supported by the Plan Project of the Science and Technology in Guizhou Province (No. Qian Ke He [2022] General 178), Guizhou Provincial Key Laboratory of Microbiome and Infectious Disease Prevention and Control (ZDSYS [2023] No. 004; URL: <https://midipc.gmc.edu.cn),> and Science and Technology Fund Project of Guizhou Provincial Health Commission (No. gzwkj2025-504; URL: <https://wjw.guizhou.gov.cn/xwzx/tzgg/202501/t20250106_86581667.html).> The funders had no role in the study design, data collection and analysis, decision to publish, or preparation of the manuscript.The funders provided financial support but had no role in study design, data collection and analysis, decision to publish, or preparation of the manuscript. We hope this satisfies the reviewer's concern.

Reviewer #2:

2.1 Environmental safety and biosafety of the laboratory R-strain must be declared in the manuscript, as per institutional and global policies.

Our response: Thank you for your valuable comment. In accordance with your suggestion and following institutional and global biosafety policies, we have added a combined statement regarding “Ethics, Environmental Safety, and Biosafety” in the Materials and Methods section (subsection 2.1). This new statement explicitly declares that the laboratory-reared pyriproxyfen-resistant strain (Lab-R) was handled under strict containment measures to prevent any environmental release, and that all animal procedures were approved by the Institutional Animal Care and Use Committee. We believe this revision addresses the reviewer's concerns comprehensively.

2.2 The reason behind choosing 13 generations of *Aedes* for the study can be justified. Did author observe any blunted response beyond 13 generations or it was exponential beyond that?

Our response: Thank you for this important comment. In this study, resistance selection was terminated at the 13th generation because the resistance ratio (RR_50_) reached the predefined threshold for high resistance (≥10-fold), which is the operational criterion for successful laboratory selection of a resistant strain in our study design. Based on the exponential growth model (y = 1.4175e*^(x/2.5995)^ + 25.8241, R^2^ = 0.9952), we estimated that the RR_50_ would continue to increase beyond the 13th generation without reaching an obvious plateau within the observed range. Nevertheless, we acknowledge that further selection beyond 13 generations was not performed, and whether the resistance response would eventually blunt or remain exponential remains unknown. This represents a limitation of the present study and warrants future investigation.

2.3 Authors may wish to provide a contrast with other species of mosquito in the discussion part and provide an elaborate account of several previous studies which have been conducted on similar lines.

Our response: Thank you for this valuable suggestion. In response, we have added a concise comparative discussion in the Discussion section (see the highlighted section in light green). Specifically, we now contrast our findings with previous studies in *Anopheles gambiae*, *Culex quinquefasciatus*, and *Aedes aegypti*, highlighting that metabolic resistance (especially P450-mediated) is a common but species-dependent mechanism underlying pyriproxyfen-pyrethroid cross-resistance. This addition improves the contextualization of our results within the broader vector biology literature.

2.4 While performing the metabolic studies authors checked the GST levels, however, it is also important to test and assess the availability and ratio of GSH:GSSG to completely rely and draw inference on GST activity . It is therefore suggested to state if the GSH /GSSG levels were also affected. Moreover, a direct microscopic examination of radicals could also provide an evidence of metabolic perturbations caused with or without insecticides.

Our response: Thank you for the reviewer's insightful comment. We acknowledge that measuring GSH:GSSG ratios and performing direct microscopic examination of radicals could provide additional information on metabolic perturbations. However, given that our current study focused primarily on the overall activity of detoxification enzymes (including GSTs) and the associated fitness costs and cross-resistance phenotypes, we did not include these specific analyses. We believe that the GST activity data, in combination with synergist tests and enzyme assays for P450s and Car Es, sufficiently support our conclusion regarding metabolic resistance. We hope the reviewer finds our response acceptable.

2.5 It is intresting to note the synergy of pyrethroid insecticides treatment, yet a plausible reason or mechanism remains unexplored, which could further strengthen the manuscript.

Our response: Thank you for this valuable comment. In the revised manuscript, we have added a plausible mechanistic explanation for the observed synergy: the enhanced activity of detoxification enzymes (P450s, GSTs, and Car Es) in the pyriproxyfen-resistant strain likely facilitates the metabolism of pyrethroids, and the addition of enzyme inhibitors (PBO, DEM, TTP) reverses this effect. This suggests that cross-resistance is metabolically driven. Relevant text has been added in the Discussion section (see the highlighted section in light green).

2.6 ''…….listed among the world's most dangerous invasive species 12'' : Probably the number 12 should be [1,2] as it indicate the reference no. Similarly in line …….chikungunya virus, posing a severe threat to public health 134., the number 134 should be [1,3,4] Please check elsewhere and correct the reference accordingly (…behavioral resistance 2628; ……. insecticides 2930.) I could notice these typos at many places and not listed all, please check if you use a reference manager and correct accordingly.

Our response: Thank you very much for your careful reading and valuable comments. We apologize for the typographical errors in the citation formatting. We have thoroughly checked the manuscript and corrected all instances where reference numbers were incorrectly placed (e.g., changing ''...dangerous invasive species 12'' to ''[1,2]'', ''...public health 134'' to ''[1,3,4]'', ''...behavioral resistance 2628'' to ''[26-28]'', and ''...insecticides 2930'' to ''[29,30]''). We have reviewed the entire manuscript using our reference manager (EndNote) to ensure all in-text citations are correctly formatted and placed. The corrections have been made in the revised manuscript. We appreciate your thoroughness.

Reviewer #3:

3.1 Technical details of kits used should be mentioned clearly. In the methodology sections 2.4 and 2.6, proper catalogue numbers of kits should be mentioned.

Our response: Thank you for your valuable comment. We have revised the manuscript accordingly. In the Methods section, we have now explicitly mentioned the catalogue numbers of the kits used:

Section 2.4 (Metabolic enzyme activity assay): The BCA Protein Assay Kit (Thermo Scientific, USA, Cat# YG372873) was used for protein quantification.

Section 2.6 (DNA extraction and gene mutation detection): The genomic DNA extraction kit (Beijing BioTec Biotechnology Co., Ltd., China, Cat# B010003025) was used.

These details have been added to the respective subsections and highlighted in red.. We believe this addresses the concern raised.

3.2 The resolution of the figures should be enhanced.

Our response: Thank you for your comment. We have enhanced the resolution of all figures (Fig. 1-Fig. 5) to meet the journal's requirements. The revised figures are now provided as high-resolution files (> 900 dpi, TIFF format) in the revised manuscript submission.

3.3 In section 4, the name of *Anopheles gambiae* should be in italics.

Our response: Thank you for your careful reading and valuable comment. We apologize for this minor oversight. In the revised manuscript, we have corrected *Anopheles gambiae* to italics in Section 4 (Discussion). The correction has been highlighted.

3.4 The study has a very strong experimental base, but as discussed in Section 4-, they have mentioned the failure to identify the specific metabolic genes and their validation at the transcriptional level. So, they can put a small experiment in which the expression analysis of these genes can be done using qRT-PCR. These results will add on the experimental data and validation.
Our response: Thank you very much for your positive assessment and constructive suggestion. We fully agree that qRT-PCR validation of specific metabolic genes would strengthen the study. However, due to current resource and time constraints, we are unable to perform additional experiments at this stage. Nevertheless, we have clearly acknowledged this limitation in the Discussion section (see the highlighted section in light green) and have proposed it as a priority for future research using transcriptomic and proteomic approaches. We hope that the existing multi-level evidence—comprising resistance selection, cross-resistance patterns, fitness costs, enzyme activity assays, synergist tests, and target gene sequencing—provides a sufficiently robust basis for the conclusions. Thank you again for your understanding.

3.5 General English language requires major revision for better clarity and grammatical errors to be corrected.

Our response: Thank you for your valuable comment. We apologize for the language issues in the manuscript. We have carefully revised the entire text to improve grammatical accuracy and clarity. We believe the language is now significantly improved and meets journal standards. A clean version of the revised manuscript is attached for your kind consideration.

We believe that the revised manuscript now fully meets the publication standards of PLOS ONE. We would like to once again thank the editor and the reviewers for their valuable time and expert feedback.

Sincerely,

Yours sincerely,

Corresponding authors: Wenqin Liang, Yong Hu

On behalf of all authors

May 25, 2026
