# Supplementary material for: Metabolic resistance drives cross-resistance in Aedes albopictus to pyriproxyfen and pyrethroid insecticides
Source: PLoS One. 2026 Aug 31;21(8):e0355196. doi: 10.1371/journal.pone.0355196 (PMC13528961; doi:10.1371/journal.pone.0355196)
Supplement: Peer review history

# Author comment: Author response to Decision Letter 0

**DOI:** 10.1371/journal.pone.0355196.r001

**Transfer Alert**

This paper was transferred from another journal. As a result, its full editorial history (including decision letters, peer reviews and author responses) may not be present.

15 Jan 2026

# Peer review: Decision Letter 0

**DOI:** 10.1371/journal.pone.0355196.r002

**Authors:** Himmat Singh

12 Apr 2026

Dear Dr. Liang,

Thank you for submitting your manuscript to PLOS ONE. After careful consideration, we feel that it has merit but does not fully meet PLOS ONE’s publication criteria as it currently stands. Therefore, we invite you to submit a revised version of the manuscript that addresses the points raised during the review process.

Please submit your revised manuscript by May 27 2026 11:59PM. If you will need more time than this to complete your revisions, please reply to this message or contact the journal office at plosone@plos.org. When you're ready to submit your revision, log on to https://www.editorialmanager.com/pone/ and select the 'Submissions Needing Revision' folder to locate your manuscript file.

- A letter that responds to each point raised by the academic editor and reviewer(s). You should upload this letter as a separate file labeled 'Response to Reviewers'.
- A marked-up copy of your manuscript that highlights changes made to the original version. You should upload this as a separate file labeled 'Revised Manuscript with Track Changes'.
- An unmarked version of your revised paper without tracked changes. You should upload this as a separate file labeled 'Manuscript'.

If you would like to make changes to your financial disclosure, please include your updated statement in your cover letter. Guidelines for resubmitting your figure files are available below the reviewer comments at the end of this letter.

If applicable, we recommend that you deposit your laboratory protocols in protocols.io to enhance the reproducibility of your results. Protocols.io assigns your protocol its own identifier (DOI) so that it can be cited independently in the future. For instructions see: https://journals.plos.org/plosone/s/submission-guidelines#loc-laboratory-protocols. Additionally, PLOS ONE offers an option for publishing peer-reviewed Lab Protocol articles, which describe protocols hosted on protocols.io. Read more information on sharing protocols at https://plos.org/protocols?utm_medium=editorial-email&utm_source=authorletters&utm_campaign=protocols.

As the corresponding author, your ORCID iD is verified in the submission system and will appear in the published article. PLOS supports the use of ORCID, and we encourage all coauthors to register for an ORCID iD and use it as well. Please encourage your coauthors to verify their ORCID iD within the submission system before final acceptance, as unverified ORCID iDs will not appear in the published article. *Only*  the individual author can complete the verification step; PLOS staff *cannot*  verify ORCID iDs on behalf of authors.

We look forward to receiving your revised manuscript.

Kind regards,

Himmat Singh

Academic Editor

PLOS One

Journal Requirements:

When submitting your revision, we need you to address these additional requirements.

1. Please ensure that your manuscript meets PLOS ONE's style requirements, including those for file naming. The PLOS ONE style templates can be found at

https://journals.plos.org/plosone/s/file?id=wjVg/PLOSOne_formatting_sample_main_body.pdf and

https://journals.plos.org/plosone/s/file?id=ba62/PLOSOne_formatting_sample_title_authors_affiliations.pdf.

2. Thank you for stating the following financial disclosure:

"Plan Project of the Science and Technology in Guizhou Province (No. Qian Ke He [2022] General 178),Guizhou Provincial Key Laboratory of Microbiome and Infectious Disease Prevention and Control (ZDSYS [2023] No. 004), Science and Technology Fund Project of Guizhou Provincial Health Commission (No. gzwkj2025-504)"

Please state what role the funders took in the study.  If the funders had no role, please state: "The funders had no role in study design, data collection and analysis, decision to publish, or preparation of the manuscript."

If this statement is not correct you must amend it as needed.

Please include this amended Role of Funder statement in your cover letter; we will change the online submission form on your behalf.

3. We note that your Data Availability Statement is currently as follows: [All relevant data are within the manuscript and its Supporting Information files.]

Please confirm at this time whether or not your submission contains all raw data required to replicate the results of your study. Authors must share the “minimal data set” for their submission. PLOS defines the minimal data set to consist of the data required to replicate all study findings reported in the article, as well as related metadata and methods (https://journals.plos.org/plosone/s/data-availability#loc-minimal-data-set-definition).

For example, authors should submit the following data:

- The values behind the means, standard deviations and other measures reported;

- The values used to build graphs;

- The points extracted from images for analysis.

Authors do not need to submit their entire data set if only a portion of the data was used in the reported study.

If your submission does not contain these data, please either upload them as Supporting Information files or deposit them to a stable, public repository and provide us with the relevant URLs, DOIs, or accession numbers. For a list of recommended repositories, please see https://journals.plos.org/plosone/s/recommended-repositories.

If there are ethical or legal restrictions on sharing a de-identified data set, please explain them in detail (e.g., data contain potentially sensitive information, data are owned by a third-party organization, etc.) and who has imposed them (e.g., an ethics committee). Please also provide contact information for a data access committee, ethics committee, or other institutional body to which data requests may be sent. If data are owned by a third party, please indicate how others may request data access.

4. Please note that funding information should not appear in the Acknowledgments section or other areas of your manuscript. We will only publish funding information present in the Funding Statement section of the online submission form. Please remove any funding-related text from the manuscript and let us know how you would like to update your Funding Statement.

5. Please include your full ethics statement in the ‘Methods’ section of your manuscript file. In your statement, please include the full name of the IRB or ethics committee who approved or waived your study, as well as whether or not you obtained informed written or verbal consent. If consent was waived for your study, please include this information in your statement as well.

6. If the reviewer comments include a recommendation to cite specific previously published works, please review and evaluate these publications to determine whether they are relevant and should be cited. There is no requirement to cite these works unless the editor has indicated otherwise.

7. Please review your reference list to ensure that it is complete and correct. If you have cited papers that have been retracted, please include the rationale for doing so in the manuscript text, or remove these references and replace them with relevant current references. Any changes to the reference list should be mentioned in the rebuttal letter that accompanies your revised manuscript. If you need to cite a retracted article, indicate the article’s retracted status in the References list and also include a citation and full reference for the retraction notice.

Additional Editor Comments (if provided):

As the study is important however, there are some observations on the Abstract lenght Justifying pyriproxyfen use as per WHO guidlines . in the methods the details for statistical methods used for enzyme activity analysis and fitness costs needs to added. modify the figures as per suggestions of the reviewers in result section and check overall MS for any repeatation.

Improve discussion and ethical statements as suggested

[Note: HTML markup is below. Please do not edit.]

Reviewers' comments:

Reviewer's Responses to Questions

**Comments to the Author**

1. Is the manuscript technically sound, and do the data support the conclusions?

Reviewer #1: Yes

Reviewer #2: Yes

Reviewer #3: Partly

**********

2. Has the statistical analysis been performed appropriately and rigorously?

Reviewer #1: Yes

Reviewer #2: Yes

Reviewer #3: N/A

**********

3. Have the authors made all data underlying the findings in their manuscript fully available?

The PLOS Data policy

Reviewer #1: Yes

Reviewer #2: Yes

Reviewer #3: Yes

**********

4. Is the manuscript presented in an intelligible fashion and written in standard English?

Reviewer #1: Yes

Reviewer #2: Yes

Reviewer #3: Yes

**********

Reviewer #1: Summary: The title is clear and concise. It accurately reflects the study's focus on metabolic resistance and cross-resistance in Aedes albopictus. The abstract effectively outlines the objectives, methods, and key findings, including resistance ratios, enzyme activity, and fitness costs.

Recommendations:

- Consider shortening the abstract to focus on the most important findings.

- More explicitly highlight the broader implications for vector control programs.

Introduction / Background

- Strengthen the justification for focusing on pyriproxyfen by connecting it more directly to WHO recommendations and recent field reports.

- Clarify how this study offers something new compared to previous work in China, Brazil, and Malaysia.

Methodology

- Provide more details on the statistical methods used for analyzing enzyme activity and fitness costs.

- Clarify how sample sizes were determined for each assay.

- Ensure ethical considerations (animal blood feeding, mouse use) are fully justified and referenced.

Results

- Present resistance ratios and enzyme activity data in clearer tables or figures for better readability.

- Discuss variability among replicates and confidence intervals.

- Consider adding statistical significance markers in the figures.

Discussion

- Expand on the implications for integrated vector management, such as insecticide rotation and synergist use.

- Compare findings with resistance mechanisms in Anopheles gambiae and other vectors to broaden relevance.

- Address limitations, including differences between laboratory strains and field populations, as well as geographic scope.

- Strengthen the call for policy and operational changes in vector control programs.

- Suggest future research directions, such as genomic studies and field validation.

Ethics, Funding, and Data Availability

- Ensure the funding statement includes grant numbers and URLs for funders.

- Confirm that funders had no role in study design or manuscript preparation.

Reviewer #2: Manuscript entitled “Metabolic Resistance Drives Cross-Resistance in Aedes albopictus to Pyriproxyfen and Pyrethroid Insecticides” is of considerable interest to the researchers in the domain of malaria biology and tropical diseases and can be useful for ongoing research. Authors have explicitly elaborated the basis of insecticide resistance, particularly for Pyriproxyfen and Pyrethroid insecticides in Ae. Albopictus.

Authors may, however, wish to answer following queries and can account for the following suggestions.

• Environmental safety and biosafety of the laboratory R-strain must be declared in the manuscript, as per institutional and global policies. Moreover, the reason behind choosing 13 generations of Ades for the study can be justified. Did author observe any blunted response beyond 13 generations or it was exponential beyond that?

• Authors may wish to provide a contrast with other species of mosquito in the discussion part and provide an elaborate account of several previous studies which have been conducted on similar lines.

• While performing the metabolic studies authors checked the GST levels, however, it is also important to test and assess the availability and ratio of GSH:GSSG to completely rely and draw inference on GST activity . It is therefore suggested to state if the GSH /GSSG levels were also affected . Moreover, a direct microscopic examination of radicals could also provide an evidence of metabolic perturbations caused with or without insecticides.

• It is intresting to note the synergy of pyrethroid insecticides treatment, yet a plausible reason or mechanism remains unexplored, which could further strengthen the manuscript.

Minor comments

“…….listed among the world's most dangerous invasive species 12” : Probably the number 12 should be [1,2] as it indicate the reference no.

Similarly in line …….chikungunya virus, posing a severe threat to public health 134., the number 134 should be [1,3,4]

Please check elsewhere and correct the reference accordingly (…behavioral resistance 2628; ……. insecticides 2930.) I could notice these typos at many places and not listed all, please check if you use a reference manager and correct accordingly.

Fig 2. Is it possible to change x- axis from “screening algebra” to “number of generation” which can help readers to comprehend better.

Reviewer #3: Comments

1. Technical details of kits used should be mentioned clearly. In the methodology sections 2.4 and 2.6, proper catalogue numbers of kits should be mentioned.

2. The resolution of the figures should be enhanced.

3. In section 4, the name of Anopheles gambiae should be in italics.

4. The study has a very strong experimental base, but as discussed in Section 4-, they have mentioned the failure to identify the specific metabolic genes and their validation at the transcriptional level. So, they can put a small experiment in which the expression analysis of these genes can be done using qRT-PCR. These results will add on the experimental data and validation.

5. General English language requires major revision for better clarity and grammatical errors to be corrected.

**********

what does this mean?). If published, this will include your full peer review and any attached files.

If you choose “no”, your identity will remain anonymous but your review may still be made public.

**Do you want your identity to be public for this peer review?**  For information about this choice, including consent withdrawal, please see our Privacy Policy

Reviewer #1: **Yes:**  Adriko Moses

Reviewer #2: **Yes:**  Aditya Arya

Reviewer #3: No

**********

[NOTE: If reviewer comments were submitted as an attachment file, they will be attached to this email and accessible via the submission site. Please log into your account, locate the manuscript record, and check for the action link "View Attachments". If this link does not appear, there are no attachment files.]

To ensure your figures meet our technical requirements, please review our figure guidelines: https://journals.plos.org/plosone/s/figures

You may also use PLOS’s free figure tool, NAAS, to help you prepare publication quality figures: https://journals.plos.org/plosone/s/figures#loc-tools-for-figure-preparation.

NAAS will assess whether your figures meet our technical requirements by comparing each figure against our figure specifications.

# Author comment: Author response to Decision Letter 1

**DOI:** 10.1371/journal.pone.0355196.r003

25 May 2026

Response to Reviewers

Dear editor and reviewers,

We have revised the manuscript and answered the editor and reviewer' s questions/comments with point by point hereunder according to the requirements in your letter.

Hence, we are re-submitting herewith the revised manuscript entitled ''Metabolic Resistance Drives Cross-Resistance in Aedes albopictus to Pyriproxyfen and Pyrethroid Insecticides'' and manuscript ID is PONE-D-26-02598.

To easily distinguish our answers from reviewer' s questions/comments, we highlighted all of our answers in blue while your letter and reviewer' s questions/comments in black in the Response letter.

Thank you again for your time and consideration. We hope the revised manuscript has now met the publication standard of your journal.

Looking forward to hearing from you.

Journal's comments:

1. Please ensure that your manuscript meets PLOS ONE's style requirements, including those for file naming.

Our response: Thank you for highlighting this. We have carefully reviewed and revised the manuscript's formatting, title, authors' affiliations, and file names in accordance with the template provided by PLOS ONE to ensure compliance with the journal's requirements.

2. Please clarify the role of funders in the study. Please note that funding information should not appear in the Acknowledgments section or other areas of your manuscript. We will only publish funding information present in the Funding Statement section of the online submission form. Please remove any funding-related text from the manuscript and let us know how you would like to update your Funding Statement.

Our response: Thank you for your feedback. We have removed all funding-related text from the manuscript, including the Acknowledgments section. The role of the funders was solely to provide financial support and they had no involvement in study design, data collection, analysis, interpretation, or manuscript preparation. Please find the updated Funding Statement as follows:

Funding Statement: This study was funded by Plan Project of the Science and Technology in Guizhou Province (No. Qian Ke He [2022] General 178), Guizhou Provincial Key Laboratory of Microbiome and Infectious Disease Prevention and Control (ZDSYS [2023] No. 004), and Science and Technology Fund Project of Guizhou Provincial Health Commission (No. gzwkj2025-504). The funders had no role in study design, data collection and analysis, decision to publish, or preparation of the manuscript.

This content has been removed from the manuscript; it has been highlighted in red and struck through.

3. Data Availability Statement.

Our response: Thank for your suggestion. All relevant data are within the manuscript and its Supporting Information files.

4. Please include your full ethics statement in the 'Methods' section of your manuscript file. In your statement, please include the full name of the IRB or ethics committee who approved or waived your study, as well as whether or not you obtained informed written or verbal consent.

Our response: We appreciate the editor's suggestion. We have added a complete ethics statement to the ‘Methods’ section (See Section 2.1 for details, text in red). This statement includes the full name of the review committee—the Animal Ethics Committee of the Guizhou Provincial Center for Disease Control and Prevention—and the approval number (DS2025-02).

5. Citations Recommended by the Reviewers.

Our response: Thank you for your feedback. The reviewer did not recommend any relevant references.

6. Completeness of the Reference List.

Our response: We are very grateful to the editor for raising this question.We have checked the reference list throughout the manuscript and confirmed that it does not contain any retracted publications.

7. As the study is important however, there are some observations on the Abstract lenght Justifying pyriproxyfen use as per WHO guidlines . in the methods the details for statistical methods used for enzyme activity analysis and fitness costs needs to added. modify the figures as per suggestions of the reviewers in result section and check overall MS for any repeatation. Improve discussion and ethical statements as suggested.

Our response: Thank you for your valuable comments on our manuscript. We have revised the manuscript accordingly.

Reviewer #1:

1.1 Consider shortening the abstract to focus on the most important findings, more explicitly highlight the broader implications for vector control programs.

Our response: Thank you for this valuable suggestion. We have simplified the abstract as suggested and included direct implications for vector control programs. Deleted text is indicated in red font with strikethrough. Added or revised text is highlighted in blue and underlined.

1.2 Strengthen the justification for focusing on pyriproxyfen by connecting it more directly to WHO recommendations and recent field reports. Clarify how this study offers something new compared to previous work in China, Brazil, and Malaysia.

Our response: We appreciate this important feedback. The introduction has been revised to incorporate recommendations from the World Health Organization (WHO) and the latest field reports on antimicrobial resistance from Brazil, Malaysia, the United States, and China, and to clarify how this study differs from previous studies conducted in China, Brazil, and Malaysia. Specific changes are highlighted in the file labeled 'Revised Manuscript with Track Changes. Deleted text is indicated in red font with strikethrough. Added or revised text is highlighted in blue and underlined.

1.3 Ensure ethical considerations (animal blood feeding, mouse use) are fully justified and referenced.

Our response: All procedures were conducted in accordance with the relevant provisions of the 'Guidelines on the Humane Treatment of Animals' and the 'Regulations on the Management of Laboratory Animals'. The ethics approval number is provided in Section 2.1 in the file labeled 'Revised Manuscript with Track Changes'.

1.4 Clarify how sample sizes were determined for each assay.

Our response: We thank the reviewer for this important question. The sample sizes in this study were determined based on established methodologies from previously published studies and international guidelines, as detailed below:

Synergist experiments and resistance development:

These experiments strictly followed the WHO guidelines for monitoring and managing insecticide resistance in Aedes mosquito populations (World Health Organization. Monitoring and managing insecticide resistance in Aedes mosquito populations. 2016.). For each concentration of insecticide (with or without synergist), three replicates were used, each containing 20 late-III to early-IV instar larvae. This sample size is the WHO-recommended standard for larval bioassays and has been widely adopted in insecticide resistance studies.

Fitness Cost Experiment:

The sample size and experimental design were primarily based on the study by Deng J et al.(Deng J, Guo Y, Su X, Liu S, Yang W, Wu Y, et al. Impact of deltamethrin- resistance in Aedes albopictus on its fitness cost and vector competence. PLoS Negl Trop Dis. 2021;15(4):e0009391.) and their corresponding thesis, which systematically assessed the fitness costs of insecticide-resistant Ae. albopictus. The sources for the experimental methods and sample size determination have been added to Section 2.4 of the manuscript. Added or revised text is highlighted in blue and underlined.

Metabolic enzyme activity assays:

The sample size and technical replicates were determined according to Tokponnon TF et al. (Tokponnon TF, Ossè R, Zoulkifilou SD, Amos G, Festus H, Idayath G, et al. Insecticide Resistance in Aedes aegypti Mosquitoes: Possible Detection of kdr F1534C, S989P, and V1016G Triple Mutation in Benin, West Africa. Insects. 2024;15(4):295.) and related studies on detoxification enzyme activity in mosquitoes. Each enzyme assay included three biological replicates per strain. This design ensures statistical reliability for enzyme activity comparisons. We have added the sample volume for the enzyme activity assay to Section 2.5 of the Methods section. Deleted text is indicated in red font with strikethrough. Added or revised text is highlighted in blue and underlined.

We have now explicitly cited these sources in the Methods section and clarified that sample sizes were based on established laboratory protocols rather than a formal power calculation, which we acknowledge as a limitation. No data-driven sample size re-estimation was performed during the study.

1.5 Provide more details on the statistical methods used for analyzing enzyme activity and fitness costs.

Our response: We thank the reviewer for this important methodological suggestion. We apologize for the lack of clarity. In the revised manuscript, we have substantially expanded the Statistical analysis section (section 2.8) to include:

Normality assessment – Shapiro-Wilk test for all continuous variables.

Variance homogeneity check – Levene's test before applying parametric tests.

Specific test choices: Two‑tailed independent t-test (homoscedastic) or Welch's t'-test (heteroscedastic) for normally distributed data; Mann-Whitney U test for non‑normally distributed data.

Survival analysis: Kaplan-Meier with log‑rank test and Mann–Whitney U for median survival.

Rate comparisons: χ² test for pupation, emergence, and hatching rates.

These changes are highlighted in the revised manuscript. Deleted text is indicated in red font with strikethrough. Added or revised text is highlighted in blue and underlined.

1.6 Present resistance ratios and enzyme activity data in clearer tables or figures for better readability.

Our response: We appreciate this constructive suggestion from the reviewers. To improve the readability of the manuscript, we have provided the raw data for the relevant figures and tables in the appendix. Please refer to the appendix for detailed results.

1.7 Discuss variability among replicates and confidence intervals.

Our response: Thank you for your careful reading and valuable comment regarding the variability among replicates and confidence intervals. In our manuscript, the variability among replicates is already reflected by the 95% confidence intervals (CI) provided for all IE50/LC50 values (Table 2, Fig 2), and standard deviations (SD) are presented for fitness cost data (Table 3, Fig 3). The number of replicates and statistical methods are described in the Materials and Methods section. Therefore, we believe the current results sufficiently convey the experimental variability without additional discussion. We kindly hope the reviewer agrees.

1.8 Consider adding statistical significance markers in the figures.

Our response: We thank the reviewer for this constructive suggestion. We would like to respectfully note that statistical significance markers (e.g., *P < 0.05, ***P < 0.0001, ns for non-significant and Identical letters within the same synergist group indicate that the difference is not statistically significant) have already been included in the relevant figures (Fig 3, Fig 4, and Fig 5) to indicate the results of statistical comparisons. To improve clarity and comply with the reviewer's expectation, we have now explicitly mentioned in the figure legends that asterisks denote statistically significant differences. We believe these revisions make the presentation of statistical results more transparent. We appreciate the reviewer's careful reading of our manuscript. Added or revised text is highlighted in blue and underlined.

1.9 Expand on the implications for integrated vector management, such as insecticide rotation and synergist use. Strengthen the call for policy and operational changes in vector control programs.

Our response: We thank the reviewer for the constructive comments. In response, we have expanded the discussion on the practical implications of our findings for integrated vector management (IVM), with a focus on insecticide rotation and the use of synergists such as PBO. We have also strengthened the policy and operational recommendations for vector control programs. For details, see the last paragraph of the discussion, deleted text is indicated in red font with strikethrough. Added or revised text is highlighted in blue and underlined.

1.10 Compare findings with resistance mechanisms in Anopheles gambiae and other vectors to broaden relevance.

Our response: Thank you very much indeed for your comment. We have added a comparative discussion on resistance mechanisms in Anopheles gambiae and other vectors (e.g., Culex quinquefasciatus, Aedes aegypti) in the Discussion section (see the highlighted section in light green). This broadens the relevance of our findings beyond Ae. albopictus and supports the conserved role of metabolic resistance across mosquito species.

1.11 Address limitations, including differences between laboratory strains and field populations, as well as geographic scope. Suggest future research directions, such as genomic studies and field validation.

Our response: We agree with the reviewer and have added the following points to the Discussion section:

Laboratory vs. field populations: We acknowledge that resistance development and fitness costs observed in a laboratory-selected strain may differ from wild populations due to genetic background and environmental factors.

Geographic scope: Our study was conducted on a single Ae. albopictus strain from one region; therefore, the findings may not be generalizable to all populations.

Future directions: We now recommend genomic studies (e.g., transcriptomics, whole-genome sequencing) to identify specific metabolic genes conferring cross-resistance, as well as field validation across multiple geographic sites to assess real-world applicability.

These revisions have been incorporated into the Discussion (see the highlighted section in light yellow) and deleted text is indicated in red font with strikethrough.

1.12 Ensure the funding statement includes grant numbers and URLs for funders. Confirm that funders had no role in study design or manuscript preparation.

Our response: Thank you for your careful and constructive comment regarding our funding statement. This work was supported by the Plan Project of the Science and Technology in Guizhou Province (No. Qian Ke He [2022] General 178), Guizhou Provincial Key Laboratory of Microbiome and Infectious Disease Prevention and Control (ZDSYS [2023] No. 004; URL: https://midipc.gmc.edu.cn), and Science and Technology Fund Project of Guizhou Provincial Health Commission (No. gzwkj2025-504; URL: https://wjw.guizhou.gov.cn/xwzx/tzgg/202501/t20250106_86581667.html). The funders had no role in the study design, data collection and analysis, decision to publish, or preparation of the manuscript.The funders provided financial support but had no role in study design, data collection and analysis, decision to publish, or preparation of the manuscript. We hope this satisfies the reviewer's concern.

Reviewer #2:

2.1 Environmental safety and biosafety of the laboratory R-strain must be declared in the manuscript, as per institutional and global policies.

Our response: Thank you for your valuable comment. In accordance with your suggestion and following institutional and global biosafety policies, we have added a combined statement regarding “Ethics, Environmental Safety, and Biosafety” in the Materials and Methods section (subsection 2.1). This new statement explicitly declares that the laboratory-reared pyriproxyfen-resistant strain (Lab-R) was handled under strict containment measures to prevent any environmental release, and that all animal procedures were approved by the Institutional Animal Care and Use Committee. We believe this revision addresses the reviewer's concerns comprehensively.

2.2 The reason behind choosing 13 generations of Aedes for the study can be justified. Did author observe any blunted response beyond 13 generations or it was exponential beyond that?

Our response: Thank you for this important comment. In this study, resistance selection was terminated at the 13th generation because the resistance ratio (RR50) reached the predefined threshold for high resistance (≥10-fold), which is the operational criterion for succ

Attachment Submitted filename: Response to Reviewers.docx

# Peer review: Decision Letter 1

**DOI:** 10.1371/journal.pone.0355196.r004

**Authors:** Sarman Singh

20 Jul 2026

<p>Metabolic Resistance Drives Cross-Resistance in Aedes albopictus to Pyriproxyfen and Pyrethroid Insecticides

PONE-D-26-02598R1

Dear Dr. Liang,

We’re pleased to inform you that your manuscript has been judged scientifically suitable for publication and will be formally accepted for publication once it meets all outstanding technical requirements and minor revisions, if any.

Within one week, you’ll receive an e-mail detailing the required amendments. When these have been addressed, you’ll receive a formal acceptance letter and your manuscript will be scheduled for publication.

An invoice will be generated when your article is formally accepted. Please note, if your institution has a publishing partnership with PLOS and your article meets the relevant criteria, all or part of your publication costs will be covered. Please make sure your user information is up-to-date by logging into Editorial Manager at Editorial Manager® and clicking the ‘Update My Information' link at the top of the page. For questions related to billing, please contact billing support.

If your institution or institutions have a press office, please notify them about your upcoming paper to help maximize its impact. If they’ll be preparing press materials, please inform our press team as soon as possible -- no later than 48 hours after receiving the formal acceptance. Your manuscript will remain under strict press embargo until 2 pm Eastern Time on the date of publication. For more information, please contact onepress@plos.org.

Kind regards,

Sarman Singh, MD, FRSC, FRCP

Academic Editor

PLOS One

Additional Editor Comments (optional):

Reviewers' comments:

Reviewer's Responses to Questions

**Comments to the Author**

Reviewer #1: All comments have been addressed

Reviewer #3: All comments have been addressed

**********

2. Is the manuscript technically sound, and do the data support the conclusions?

Reviewer #1: Yes

Reviewer #3: Yes

**********

3. Has the statistical analysis been performed appropriately and rigorously?

Reviewer #1: Yes

Reviewer #3: N/A

**********

4. Have the authors made all data underlying the findings in their manuscript fully available?

The PLOS Data policy

Reviewer #1: Yes

Reviewer #3: Yes

**********

5. Is the manuscript presented in an intelligible fashion and written in standard English?

Reviewer #1: Yes

Reviewer #3: Yes

**********

Reviewer #1: General assessment: The revised manuscript, titled “Metabolic Resistance Drives Cross-Resistance in Aedes albopictus to Pyriproxyfen and Pyrethroid Insecticides,” is well designed and contains solid experimental data. The authors responded to previous reviewer and editorial comments and have clarified the text, made it more transparent, and added more relevance.

This study fills an important knowledge gap in the field of cross-resistance in Aedes albopictus an important gap in our understanding about the role of aepidicides in vector control. The following provide good strengths to the manuscript;

•Robust experimental design: The manuscript features strong experimental design and strong experimental design with strong data for metabolic resistance through 13 generations of laboratory selection, synergist assay, and enzyme activity profile. Clarity in the paper has been clarified. The presence of statistical methods (Shapiro-Wilk, Levene’s test, Kaplan-Meier survival analysis) and a justification of sample size ensures reproducibility.

•Better contextualization: The introduction and discussion have been revised to better contextualize the results in light of WHO recommendations and compare the mechanisms of Anopheles gambiae, Culex quinquefasciatus and Aedes aegypti.

•Disclosure of ethical and biosafety: The manuscript includes information on IRB approval, animal welfare and biosafety containment.

•Policy relevance: More discussion on insecticides for different vectoricides in PBO.

•Clearities of importance: More discussion on rotation of insecticides, use of synergist (PBO) and recommendations for vector control.

•Weaknesses /Open issues: The abstract is short but should be clearer.

•Interpretation of GST activity: No GSH:GSSG ratio analysis is conducted, limiting my knowledge.

•Genomic validation: Lack of qRT-PCR or transcriptomic validation for certain metabolic genes.

Decision. Accept with minor changes.

Reviewer #3: (No Response)

**********

what does this mean?). If published, this will include your full peer review and any attached files.

If you choose “no”, your identity will remain anonymous but your review may still be made public.

**Do you want your identity to be public for this peer review?**  For information about this choice, including consent withdrawal, please see our Privacy Policy

Reviewer #1: No

Reviewer #3: No

**********

# Editor report: Acceptance letter

**DOI:** 10.1371/journal.pone.0355196.r005

**Authors:** Sarman Singh

PONE-D-26-02598R1

PLOS One

Dear Dr. Liang,

I'm pleased to inform you that your manuscript has been deemed suitable for publication in PLOS One. Congratulations! Your manuscript is now being handed over to our production team.

At this stage, our production department will prepare your paper for publication. This includes ensuring the following:

* All references, tables, and figures are properly cited

* All relevant supporting information is included in the manuscript submission,

* There are no issues that prevent the paper from being properly typeset

You will receive further instructions from the production team, including instructions on how to review your proof when it is ready. Please keep in mind that we are working through a large volume of accepted articles, so please give us a few days to review your paper and let you know the next and final steps.

Lastly, if your institution or institutions have a press office, please let them know about your upcoming paper now to help maximize its impact. If they'll be preparing press materials, please inform our press team within the next 48 hours. Your manuscript will remain under strict press embargo until 2 pm Eastern Time on the date of publication. For more information, please contact onepress@plos.org.

You will receive an invoice from PLOS for your publication fee after your manuscript has reached the completed accept phase. If you receive an email requesting payment before acceptance or for any other service, this may be a phishing scheme. Learn how to identify phishing emails and protect your accounts at https://explore.plos.org/phishing.

If we can help with anything else, please email us at customercare@plos.org.

Thank you for submitting your work to PLOS ONE and supporting open access.

Kind regards,

PLOS ONE Editorial Office Staff

on behalf of

Professor Sarman Singh

Academic Editor

PLOS One
